# Two Cases of Maggot-Associated *Ignatzschineria* Bacteremia in Xylazine-Induced Injection Wounds: An Emerging Threat

**DOI:** 10.1155/crdi/7684187

**Published:** 2024-12-10

**Authors:** Erin Pomerantz, Olivia Pericak, Carly Sokach, Jocelyn Edathil, Ho-Man Yeung

**Affiliations:** Department of Medicine, Temple University Hospital, Philadelphia, Pennsylvania, USA

**Keywords:** fentanyl, *Ignatzschineria* bacteremia, injection drug use, maggots myiasis, xylazine wound

## Abstract

The city of Philadelphia has seen an increase in homelessness and substance use disorders, often associated with xylazine-contaminated opiates. Here, we report the first two cases of wound infection and bacteremia associated with the Gram-negative rod *Ignatzschineria* species. Both cases were associated with maggot colonization in chronic lower extremity wounds from fentanyl/xylazine injections. Poor living conditions and lack of wound care are central to both case presentations. We believe this organism to be an emerging medical threat associated with injection drug use, xylazine-associated wounds, and homelessness which may impact future treatment options in this patient population. This report underscores the emergence of *Ignatzschineria* bacteremia in individuals with a history of xylazine-associated wounds and substance use disorder. Successful management should prioritize wound care and adherence to antibiotic regimens to prevent complications in these challenging cases.

## 1. Introduction

The ongoing opioid epidemic continues to inflict deleterious consequences on patients with opiate use disorder. After the rise of fentanyl, a substance known as xylazine (tranq) is often incorporated into the supply to enhance sedative effects. Xylazine is an alpha-2-adrenergic agonist commonly used as a veterinary anesthetic and sedative [1]. Although not approved for human use, xylazine is causing detrimental health concerns in persons who inject drugs (PWID) including overdose and wound-related infection. The exact mechanism for xylazine-induced skin injury is unclear, but it has been thought to be due to peripheral vasoconstriction leading to vascular damage. Other postulated causes of skin injury include cytotoxic effects, high frequency of injection, pressure injury from prolonged sedation, and small vessel vasculopathy. Although human studies are limited, animal models have shown xylazine to be responsible for decreased tissue oxygen perfusion, which can contribute to poor wound healing [2]. These wounds can occur at injection sites and noninjection sites of the skin. Wound-related infection is common due to poor living conditions, minimal access to healthcare, and inadequate hygiene [3–5].

Here, we report two separate cases of atypical bacteremia caused by the Gram-negative rod *Ignatzschineria* species from maggot-infested xylazine wounds during the summer season in Philadelphia, PA. To our knowledge, this is the first report of *Ignatzschineria* bacteremia in patients with myiasis of xylazine wounds. Given the current status of the opioid epidemic, we believe that *Ignatzschineria* is an emerging threat to populations with high rates of both homelessness and use of xylazine-containing substances. The Philadelphia Department of Public Health documented 1413 unintentional overdose deaths in 2022, indicating an increasing trend compared to previous years, with 34% of these overdose fatalities linked to xylazine involved in the use of fentanyl [5]. Philadelphia, in particular, faces challenges including alternate drug supply, significant injuries resulting from xylazine use, stimulant-related overdoses, overdoses among individuals not identifying as substance users, and the widespread impact of this crisis throughout the city, particularly affecting communities of color [5]. Additionally, xylazine also suppresses respiration which makes overdose reversals more difficult [5]. Various cases of xylazine wound-related bacteremia have been previously reported in Philadelphia with typical causative organisms including *Staphylococcus* and *Streptococcus*. *Ignatzschineria* bacteremia has been reported previously but never in association with wounds from xylazine.

## 2. Case Presentations

### 2.1. Case 1

**Figure 1:**
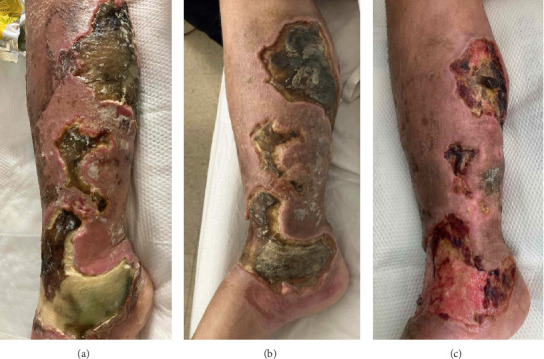
Photos from Case 1: chronic xylazine wound of the right lower extremity on initial presentation (a), 1 month later (b), and at 3 months (c). Images were taken after maggot removal.

A 38-year-old female with injection drug use, hepatitis C, HIV, and bipolar disorder, presented with a right leg wound infested with maggots. Her right lower extremity showed an ulcerated, eschar-appearing wound surrounded by erythema and induration that was tender to palpation (Figure 1). The patient reported intravenous use of 2.5 bundles (approximately 30 bags) of fentanyl/xylazine and 3 bars (6 mg) of alprazolam daily. The patient has used substances for 9 years and maintained her addiction through sex work. She reports homelessness with safety, food, and financial insecurities. Labs revealed a normal WBC count (7.1 k/μL) and elevated CRP (34.0 mg/L). CT of the lower right extremity showed cutaneous and subcutaneous ulcerations with localized pockets of soft tissue gas deep to the ulceration, diffuse cellulitis, and an abscess in the subcutaneous soft tissue of the dorsal foot at the level of the second and third metatarsal bases. She was managed with local wound care, bedside debridement, and antibiotic therapy with vancomycin and piperacillin/tazobactam. Prior to susceptibility result, she self-discharged on hospital day 3, with prescriptions for doxycycline and amoxicillin/clavulanic acid. Blood cultures later grew *Ignatzschineria ureiclastica*, susceptible to piperacillin/tazobactam, cefepime, ceftazidime, meropenem, aztreonam, ciprofloxacin, gentamicin, and trimethoprim/sulfamethoxazole. The patient returned 1 month later but left again after 5 days. Two weeks later, she returned with the same complaint and received 10 days of amoxicillin/clavulanic acid and trimethoprim/sulfamethoxazole. She declined inpatient drug rehab and was ultimately lost to follow-up.

### 2.2. Case 2

**Figure 2:**
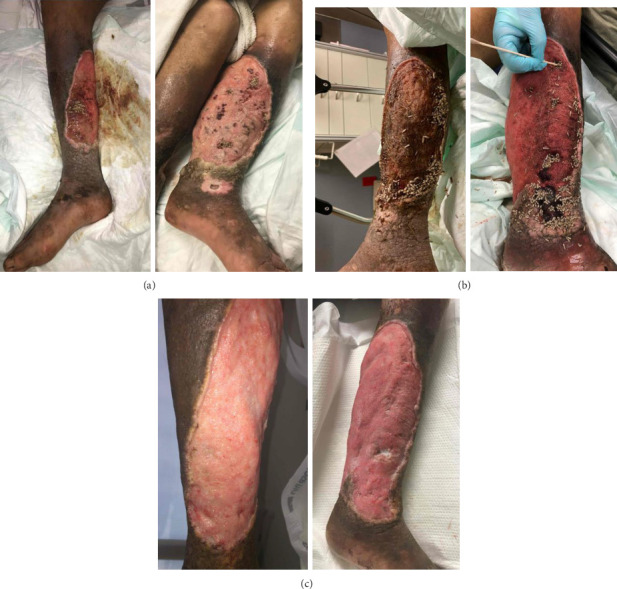
Photos from Case 2: taken upon initial presentation showing myiasis of bilateral lower limb wound (a), readmission 1 month later with recontamination (b), and prior to discharge (c).

The second case is a 46-year-old male with injection drug use who presented with maggot infestation and drainage of his wounds. He currently injects into his legs, but previously “skin popped” into his neck. He reported using 1-2 bundles of fentanyl (approximately 20 bags) daily and has been using opioids for 20 years. He is homeless and dwells in the community, lacking social support. Last hospitalization was 1 month prior for cellulitis, myositis and osteomyelitis of the lower extremities, accompanied by maggot infestation, treated with vancomycin and cefepime. Blood cultures during that admission revealed *Wohlfahrtiimonas chitiniclastica*, a gram-negative bacillus found in the larva of *Wohlfahrtia magnifica*. He returned due to progressive lower extremity pain. On physical examination, the patient had generalized leg pain with maggots in bilateral leg wounds and dark-colored discharge from his left leg (Figure 2). Labs revealed a WBC count of 10 K/μL. CT confirmed cellulitis with soft tissue defects along the anteromedial aspect of the lower legs, and pockets of entrapped superficial soft tissue gas, in addition to bilateral tibial osteomyelitis, and prominent inguinal and femoral lymphadenopathy. He was empirically started on daptomycin and cefepime. Dakin's Solution was applied to cleanse the wound. Blood cultures grew *Moraxella* species, *Ignatzschineria larvae* and *Alkaliphilus oremlandii. Ignatzschineria* was susceptible to piperacillin/tazobactam, cefepime, ceftazidime, meropenem, aztreonam, ciprofloxacin, gentamicin, and trimethoprim/sulfamethoxazole. *Alkaliphilus oremlandii* was susceptible to penicillin, piperacillin/tazobactam, ertapenem, clindamycin, and metronidazole. On day 3 of admission, all maggots were removed, necrotic tissues were debrided at bedside and his wound was dressed with Dakin's Solution soak. The patient reported improved lower extremity pain the following day. He subsequently eloped from the hospital without any prescriptions, but returned to the hospital 2 months later after bystanders found him unconscious. During that admission, his wounds were infested with maggots again and he had *Pasteurella* bacteremia. Ultimately, he left the hospital again prior to completion of treatment and was lost to follow-up.

## 3. Discussion

The *Ignatzschineria* genus is a gram-negative bacterium that is nonmotile, nonsporulating, nonhemolytic, and rod-shaped [6]. The species belonging to this genus are*: Ignatzschineria indica*, *I. ureiclastica*, and *I. larvae*, first isolated from the intestinal tract of *Wohlfahrtia magnifica* larvae [7]. This case report presents two different patients of *Ignatzschineria* bacteremia associated with myiasis in Philadelphia, Pennsylvania within 2 months during the summer season. Both cases can be attributed to poor living conditions, ongoing intravenous drug use, and chronic xylazine-associated wounds. While myiasis infections are mostly documented in tropical and subtropical environments, strong social risk factors predispose patients to *Ignatzschineria* infections even in urban settings. These cases are particularly challenging given the extent of xylazine wounds. As illustrated in Figures 1 and 2, xylazine-associated wounds are profoundly advanced and require intensive wound care. In addition to *Ignatzschineria*, other uncommon organisms hosted by the larvae of *Wohlfahrtia magnifica* such as *Wohlfahrtiimonas* may be seen on cultures.

Other cases of *Ignatzschineria* bacteremia have rarely been reported. The first reported case of *Ignatzschineria* bacteremia occurred in 2014 [7]. To our knowledge, there are a total of 17 other reports in the literature (summarized in Table 1) [7–24]. All these cases described patients with chronic wound myiasis and none are associated with injection drug use or xylazine. Only one case resulted in death, however, there was “no evident cause” reported [8]. Compared to other reported cases of *Igantzschineria* bacteremia, typical wounds associated with diabetes, peripheral vascular disease and other conditions compromise the vascular supply and the inflammatory response, which make these individuals more susceptible to skin infections. Drawing parallel to those conditions, wounds from recurrent xylazine exposure may share a similar mechanism of reduction of blood supply and damage to small vessels, leading to diminished oxygen delivery and causing soft tissue infections and delayed healing process. Necrotic tissues attract parasitic fly for which larvae can feed on, leading to maggot infestation, exposing the human host to *Ignatzschineria* [25]. This was further exacerbated by social factors and environmental factors, such as hot summer temperature.

Poor living conditions can lead to significant barriers in accessing medical care such as lack of transportation, inability to pay for medical care, and competing priorities such as finding food and shelter. Addressing the challenges in treating these presentations of wounds, especially those infected with uncommon organisms, requires medical interventions in addition to a comprehensive approach considering the patients' psychosocial factors that are critical in determining treatment and care. For patients that choose to leave the hospital, managing the treatment of their infections becomes more challenging. Physicians must balance the patient's health with incomplete work-up, adherence to therapy, and antimicrobial resistance.

Implementing preventative measures and providing trauma-informed care to patients can prevent limb complications and recurrent bacteremia cases. Particularly in patients who are already presenting with severe extremity wounds, emphasis should be placed on seeking early treatment to prevent advanced wound degradation processes. In addition, counseling and resources related to addiction recovery should be provided with each encounter.

## 4. Conclusion

In summary, *Ignatzschineria* bacteremia in PWID should be considered as an emerging threat as maggot infestations of wounds become more common. Careful consideration should be emphasized on proper wound care and adherence to antibiotic regimens to prevent further complications arising from these wounds. Moreover, prevention and trauma-informed care are essential to reducing potential complications associated with wound infections. This is the first report of *Ignatzschineria* bacteremia in patients with myiasis of xylazine wounds. These cases underscore the importance of both proficient wound recuperation techniques and strict adherence to antibiotic therapy.

## Figures and Tables

**Table 1:** Summary of cases in the literature between 2014 and 2023.

Author	Year	Location	Blood cultures	Relevant information	Treatment	Outcome
Barker et al. [6]	2014	United States	*Ignatzschineria indica*	Homelessness, chronic left foot wound	Wound debridement, left third toe amputation, empiric ampicillin-sulbactam/vancomycin narrowed to cephalexin	Lost to follow up
	2014	United States	*Streptococcus pyogenes* *Ignatzschineria indica*	Chronic alcohol use, chronic left heel ulcer wound	Left leg amputation, empirici piperacillin-tazobactam/clindamycin narrowed to vancomycin/ciprofloxacin	
	2014	United States	*Ignatzschineria indica*	Paraplegic, poor hygiene, chronic decubitus ulcers		

Le Brun et al. [7]	2015	France	*Enterococcus faecalis* *Enterobacter cloacae* *Providencia stuartii* *Corynebacterium* spp. *Ignatzschineria* spp.	Cardiac arrest, necrotic wound of right shoulder, maggots around genitalia	ICU admission (had cardiorespiratory arrest), empiric ceftriaxone	Expired

Heddema, Janssen, and van Westreenen [8]	2016	Netherlands	*Ignatzschineria* spp.	Chronic alcohol use, right foot wounds, loss of consciousness	ICU admission for noninvasive ventilation, amoxicillin-clavulanic acid	Discharged

Cipolla et al. [10]	2017	Argentina	*Ignatzschineria indica*	Homelessness, chronic alcohol use, necrotic ulcer of the left lower extremity	Debridement, left leg amputation, ciprofloxacin/clindamycin	Discharged

Muse et al. [11]	2017	United States	*Streptococcus gallolyticus* *Streptococcus anginosus* *Ignatzschineria indica*	Cardiac arrest, bedbound status, sacral decubitus ulcer, left heel ulcer, laceration of the back	ICU admission requiring vasopressors, mechanical ventilation. Wound debridement. Empiric vancomycin, cefepime and metronidazole, narrowed to levofloxacin	Discharged

Rodríguez-Zúñiga et al. [12]	2018	Spain	*Ignatzschineria indica*	Chronic alcohol use, wounds on right lower leg and left foot	Amoxicillin-clavulanic acid	Discharged

Nadrah et al. [13]	2019	Slovenia	*Ignatzschineria* spp. *Enterobacter cloacae* MRSA	Irregular migrant, poor hygiene, diffuse bilateral superficial wounds on thighs and legs	Serial wound debridement, empiric flucloxacillin/ciprofloxacin, then imipenem/cilastatin/vancomycin	Discharged to detention center. Unable to follow up

Grasland et al. [14]	2019	France	*Ignatzschineria larvae*		Ceftriaxone/gentamicin narrowed to amoxicillin-clavulanic acid	

Deslandes et al. [15]	2019	Canada	*Ignatzschineria indica*	Poor hygiene, diabetes, chronic left leg wound	Empiric piperacillin-tazobactam, narrowed to amoxicillin/clavulanic acid	Discharged

Lysaght et al. [16]	2020	United States	*Wohlfahrtiimonas chitiniclastica* *Ignatzschineria indica* *Providencia stuartii*	Chronic lymphedema, left foot, and leg ulcers	Debridement, empiric vancomycin, clindamycin and piperacillin-tazobactam, narrowed to cefepime	Discharged

Snyder, Singh, and Goldman [17]	2020	United States	*Wohlfahrtiimonas chitiniclastica* *Ignatzschineria indica* MRSA	Poor hygiene, peripheral vascular disease, chronic bilateral leg wound	Empiric vancomycin/cefepime, switched to daptomycin/ceftriaxone	Discharged

Berthod et al. [18]	2020	Switzerland	*Ignatzschineria larvae*		Amoxicillin/clavulanic acid, transitioned to trimethoprim/sulfamethoxazole	

Fear et al. [19]	2020	Canada	*Ignatzschineria indica*	Homelessness, diabetes, gangrenous wound of the left leg	Empiric piperacillin-tazobactam narrowed to amoxicillin-clavulanate	Discharged

Do et al. [20]	2021	United States	*Ignatzschineria* spp. Group C/G beta *Streptococcus* *Providencia stuartii*	Poor hygiene, chronic wound on anterior left ankle	Irrigation and debridement. Empiric vancomycin, cefepime, changed to piperacillin-tazobactam	Discharged

Maniam and Argentine [21]	2021	United States	*Ignatzschineria* spp.	Opioid use disorder, peripheral vascular disease, poor hygiene, chronic left foot wound	Empiric vancomycin/cefepime, narrowed to levofloxacin	Discharged

Reed, Reynolds, and Smith [22]	2021	United States	*Klebsiella pneumoniae* *Ignatzschineria* spp.	Opioid use disorder, cocaine use disorder, self-neglect, left leg wound	Piperacillin-tazobactam and then meropenem	Incomplete treatment, lost to follow-up

DiFranza et al. [23]	2021	United States	*Ignatzschineria* spp. MRSA	Opioid used disorder, chronic alcohol use, poor hygiene, chronic venous stasis with left leg ulcerated wound	Tazobactam/vancomycin, narrowed to levofloxacin/doxycycline	Discharged

Demurtas, Pareti, and Madanchi [24]	2023	Switzerland	*Ignatzschineria larvae*	Poor hygiene, peripheral vascular disease, chronic bilateral leg ulcers	Wound debridement, ertapenem, narrowed to trimethoprim/sulfamethoxazole	Discharged

## Data Availability

Data are available upon request to corresponding author.
